# Two hundred and fifty-four metagenome-assembled bacterial genomes from the bank vole gut microbiota

**DOI:** 10.1038/s41597-020-00656-2

**Published:** 2020-09-23

**Authors:** Anton Lavrinienko, Eugene Tukalenko, Timothy A. Mousseau, Luke R. Thompson, Rob Knight, Tapio Mappes, Phillip C. Watts

**Affiliations:** 1 Department of Biological and Environmental Science, University of Jyväskylä, 40014 Jyväskylä, Finland; 2 National Research Center for Radiation Medicine of the National Academy of Medical Science, Kyiv, 04050 Ukraine; 3 Department of Biological Sciences, University of South Carolina, Columbia, SC 29208 USA; 4 School of Biological, Environmental, and Earth Sciences and Northern Gulf Institute, University of Southern Mississippi, Hattiesburg, Mississippi USA; 5 Ocean Chemistry and Ecosystems Division, Atlantic Oceanographic and Meteorological Laboratory, National Oceanic and Atmospheric Administration, Miami, Florida USA; 6 Department of Pediatrics, University of California San Diego, La Jolla, CA 92037 USA; 7 Department of Computer Science and Engineering, University of California San Diego, La Jolla, CA 92037 USA; 8 Center for Microbiome Innovation, University of California San Diego, La Jolla, CA 92037 USA

**Keywords:** Bacterial genomics, Microbial ecology, Sequencing, Metagenomics

## Abstract

Vertebrate gut microbiota provide many essential services to their host. To better understand the diversity of such services provided by gut microbiota in wild rodents, we assembled metagenome shotgun sequence data from a small mammal, the bank vole *Myodes glareolus* (Rodentia, Cricetidae). We were able to identify 254 metagenome assembled genomes (MAGs) that were at least 50% (*n* = 133 MAGs), 80% (*n* = 77 MAGs) or 95% (*n* = 44 MAGs) complete. As typical for a rodent gut microbiota, these MAGs are dominated by taxa assigned to the phyla Bacteroidetes (*n* = 132 MAGs) and Firmicutes (*n* = 80), with some Spirochaetes (*n* = 15) and Proteobacteria (*n* = 11). Based on coverage over contigs, Bacteroidetes were estimated to be most abundant group, followed by Firmicutes, Spirochaetes and Proteobacteria. These draft bacterial genomes can be used freely to determine the likely functions of gut microbiota community composition in wild rodents.

## Abstract (summary)

**Table:** 

Measurement(s)	genome • DNA • gut microbiome measurement • metagenomic data
Technology Type(s)	DNA sequencing
Sample Characteristic - Organism	Bacteroidetes • Firmicutes • Spirochaetes • Proteobacteria • Myodes glareolus

Machine-accessible metadata file describing the reported data: 10.6084/m9.figshare.12936797

## Background & Summary

Vertebrate gut microbiota are often complex communities^1,2^ that are important determinants of their host’s health^3,4^, by providing essential nutrients and metabolites^5,6^, modulating the host’s immune system^7^ and by limiting the niche space available for colonisation by pathogens^8,9^. Laboratory rodents have provided compelling evidence that the gut microbiota has an important role in maintaining host health^10,11^. The diversity of species within a gut microbiota provides the host with potential access to thousands of novel, accessory genes^1^. Understanding the type and diversity of services that can be delivered by the gut microbiota to the host requires knowledge about microbial genomes.

Gut microbiota composition of wild rodents have been characterised using marker gene sequencing surveys. Within the superfamily Muroidea (containing mice, rats, voles, hamsters, gerbils and related taxa)^12^, for example, gut microbiota of wild caught mice *Mus musculus domesticus* are dominated by the bacterial phyla Firmicutes and Bacteroidetes^13–17^. Wild wood mice *Apodemus sylvaticus* show marked seasonal variation in gut microbiota, presumably reflecting a change in diet^14^; other studies have found an association between helminth infection and gut microbiota community in wild mice *Apodemus flavicolus*^13^. Moreover, the gut microbiota of bank voles *Myodes glareolus* (arvicoline voles within the Cricetidae) may be altered by anthropogenic environment impacts. For example, the gut microbiota of *M. glareolus* inhabiting areas contaminated by radionuclides (adjacent to the former nuclear power plant at Chernobyl, Ukraine) were characterised by an increase in Firmicutes and a reduction in Bacteroidetes^16,17^. Exposure to radionuclides is also associated with a reduction in (1) inter-individual variation and (2) the degree of temporal changes in bank vole gut microbiota community composition^18^.

While a change in microbiota composition can elicit a change in its function^6^, the impact of altered gut microbiota community composition in wild rodents is unknown because available genome/gene catalogue information is biased towards microbiota derived from laboratory animal models^19,20^ rather than wild animals. To better understand whether a change in gut microbiota could have some functional relevance, we used shotgun sequencing to construct metagenome assembled genomes (MAGs) for the gut microbiota of an arvicoline rodent, the bank vole *M. glareolus*. Bank voles are common in forest habitats of central and northern Europe and parts of Asia^21^. Also, bank voles are used as a model species in evolutionary ecology research^22,23^, for example with selection lines created to examine effects of physiology and diet on gut microbiota^24^.

**Fig. 1:**
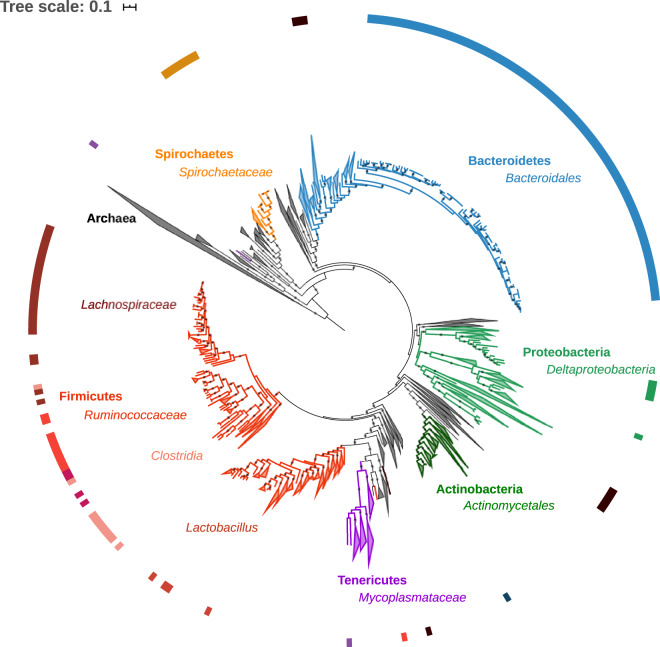
Phylogenetic diversity of metagenome assembled genomes (MAGs) from bank vole *Myodes glareolus* gut microbiota and reference genomes of Bacteria and Archaea available in RefSeq (listed in Supplementary Table 4). Presence of a MAGs from the bank vole gut microbiota is highlighted by the coloured outer ring, with black indicating the MAGs whose taxonomy was not be resolved (to phyla or better resolution) using either ANVI’O or CheckM. Midpoint rooted maximum likelihood tree was constructed from the concatenated alignments of 16 ribosomal proteins. Size of grey circles on branch midpoints indicate the level of bootstrap support (range 0.8–1.0).

We generated shotgun sequence data for DNA extracted from the faeces of bank voles. Here we present a description of 254 draft bacterial genomes (MAGs) that we estimate to be 50% or more complete and with ≤10% contamination. Phylogenetic analysis of these MAGs indicates that they comprise a typical gut microbiota for rodents within the superfamily Muroidea^13–17^, being dominated by members of the Bacteroidetes (*n* = 132) and Firmicutes (*n* = 80), but with some Spirochaetes (*n* = 15) and Proteobateria (*n* = 11) (Fig. 1). Bacteroidetes were estimated to be most abundant (55% abundance based on coverage, with all taxa assigned to the Bacteroidales), followed by Firmicutes (24%, dominated by MAGs assigned to Clostridiales), Spirochaetes (12%, all MAGs assigned to Spirochaetales) and Proteobacteria (3%, predominantly Desulfovibrionales) (Table 1). Taxonomic placements for each MAG, as well as contig statistics and estimates of genome completeness and MAG abundance, are provided in Supplementary Table 3.

**Table 1:** Relative proportion of phyla and families of bacteria present in the bank vole *Myodes glareolus* gut microbiota; na indicates unassigned taxonomic classification.

Phylum	Proportion	
*Family*		
**Actinobacteria**	0.94	
*Actinomycetales*		0.94
**Bacteroidetes**	54.90	
*Bacteroidales*		54.90
**Firmicutes**	23.93	
*Clostridiales*		21.34
*Erysipelotrichales*		0.11
*Lactobacillales*		1.84
*na*		0.64
**Proteobacteria**	3.05	
*Campylobacterales*		0.84
*Desulfovibrionales*		2.21
**Spirochaetes**	12.10	
*Spirochaetales*		12.10
**Tenericutes**	0.37	
*Mycoplasmatales*		0.37
**(na Bacteria)**	(4.71)	

These draft bacterial genomes to provide a useful resource to quantify the functions of the dominant members of wild rodent gut microbiota. Moreover, given the emerging interest in identifying the functions of relatively uncharacterised families of bacteria, such as the S24-7 that often dominate rodent gut microbiota^25,26^, these data should facilitate comparative genomic analyses of gut microbiota function and evolution across mammalian hosts. Also, the bank vole is a hyper-reservoir of zoonotic pathogens^27–30^. Given its role as a reservoir host of zoonotic pathogens, the potential link between gut microbiota and host health^3,4^, and evidence that infections associate with altered gut microbiota in rodents^13,14^, these data can provide detailed insights into the potential changes in services provided by gut microbiota that accompany infections in the bank vole. Draft genomes are deposited with NCBI Genbank under the BioProject accession PRJNA613381^31^, with metadata about the (1) bank vole samples and the (2) MAGs provided under the BioSample accessions SAMN14404158-SAMN14404199 and SAMN14407068-SAMN14407322 respectively.

## Methods

### Sampling and read data collection

Two faecal samples from 20 bank voles were collected from within the Chernobyl Exclusion Zone, Ukraine (51.30 N, 30.07 E) during May-June (faecal sample 1) and June-July 2016 (faecal sample 2). Full details of the live trapping procedures are provided in Lavrinienko *et al*.^16,17^. All procedures were performed in accordance with legal requirements and regulations from the Ukrainian authorities (957-i/16/05/2016) and the Animal Experiment Board in Finland (ESAVI/7256/04.10.07/2014). Metadata (*e.g*. location, body size, weight) associated with these samples are provided in Supplementary Table 1. Samples were transported (on dry ice) to Finland for DNA extraction based on the import permission from the Evira (3679/0460/2016). Total DNA was isolated from 0.1 g of faecal material using a PowerFecal DNA Isolation kit (MoBio Laboratories, Carlsbad, CA, USA) following the manufacturer’s instructions. Briefly, DNA extractions were performed in a dedicated laboratory space within a laminar flow hood using aseptic techniques (surface sterilisation, sterile plastic, aerosol barrier filter tips). We did not extract blank DNA samples as this procedure returns insufficient DNA for library construction and faecal samples contain high microbial biomass; rather we extracted DNA from two samples twice to assess to confirm that a sample’s microbiota profile was consistent (see Technical Validation). All the procedures were completed within 10 days by AL, using the same DNA isolation kit batch for all the samples (Supplementary Table 1). DNA concentrations were quantified with a Qubit 2.0 fluorometer (Invitrogen, Carlsbad, CA, USA) (data provided in Supplementary Table 2).

NGS data were generated using Illumina HiSeq 4000 sequencing technology to generate 100 bp paired end read data at the Beijing Genomics Institute (BGI, www.bgi.com/global/). Read data are available at NCBI Genbank (SRA) under the accession numbers SRR11425428-SRR11425469 (Supplementary Table 1), and within the BioProject accession number PRJNA613381^31^.

### Assembly

Read data were processed using ATROPOS^32^ v.1.1.5 (parameters: -q 15 --minimum-length 90), after which the reads were mapped (BOWTIE2^33^ v.2.3.4, parameters: --very-sensitive) against a draft bank vole genome (GCA_001305785.1) to filter out reads (from *.bam files) derived from the host (SAMTOOLS v.1.4 view, parameters: view -f 12 -F 256) (https://www.htslib.org/doc/samtools.html). Assembly of metagenome read data to obtain draft bacterial genomes followed the approach used to recover draft genomes from the TARA oceans metagenomics data^34^. Individual samples were assembled using MEGAHIT^35^ v.1.1.1-2 (parameters: default) to reduce memory requirements and in attempt to avoid bubbles (unresolvable branches) due to genetic diversity among strains. Using MEGAHIT, we assembled a total of 1,057 million paired end reads into 4,721,549 primary contigs (5,916,721,003 bp). These primary contigs were filtered to retain only those contigs ≥ 2 kbp in length, which were then passed through CD-HIT-EST^36^ v.4.7.0 (parameters: -c 0.99 -n 11 -M 0) to merge the completely overlapping contigs (at 99% identity); this reduced set of primary contigs was then co-assembled using MINIMUS2 in AMOS^37^ v.3.1.0 (parameters: -D REFCOUNT = 0 OVERLAP = 100 MINID = 95) to combine overlapping contigs. After this procedure, we were left with 171,806 secondary contigs (39,092 contigs and 132,714 singletons) for binning.

### Construction of metagenome assembled genomes (MAGs)

Host-filtered metagenomic reads were mapped against the secondary contigs using BOWTIE2^33^ v.2.3.4 (parameters: --sensitive). Binning of contigs into MAGs was competed using BINSANITY^38^ v.0.2.7. First, we generated a coverage file using BINSANITY profile (parameters: scale; multiply by 100 and log transform). We then ran the secondary contigs through BINSANITY-LC workflow (parameter: -x 5000 -C 50 -p -5); use of a 5 kbp length cutoff for contigs (which meant that 66,480 secondary contigs were input into BINSANITY) and the BinSanity-lc script was required as there were too many contigs to complete the binning procedure using BinSanity-wf and/or all secondary contigs. BinSanity-lc attempts to overcome memory limitation problems associated with binning many contigs by creating subsets of contigs using K-means clustering prior to implementing affinity propagation for clustering. The quality and likely taxonomic identities of the putative MAGs was assessed using two softwares^39–42^. First, we used CHECKM^39^ that searches for the occurrence of a collection of lineage-specific markers genes in the putative bins. Second, we mapped read data to the contigs (constructed by MEGAHIT) using BOWTIE2^33^ (parameters: --sensitive --no-unal), and the resulting *.sam files were processed using the metagenomic workflow implemented by ANVI’O v.5.2^40,41^: ANVI’O also assesses completeness and contamination of MAGs by searching for presence of a collection of 139 bacterial single-copy core genes^42^. Bins were defined as ‘high completion’ when they met the following criteria with either ANVI’O or CHECKM: ≥95% complete with ≤10% redundancy (category 1), ≥80% complete with ≤5% redundancy (category 2), or ≥50% complete with ≤2% redundancy (category 3). ANVI’O (--anvi-summarize, parameters: default) was used to estimate the relative proportions of bacterial phyla and families based on the average coverages of MAGs (that had been divided by the overall average coverages in samples) (Table 1).

Using BINSANITY, contigs were placed into 900 bins, of which 775 (86%) contained putative bacterial DNA (based on the presence of the core panel of markers used by CHECKM). Two hundred and fifty-four (33%) of these bins were classified as high-quality bacterial metagenomes (MAGs), representing 16,395 contigs (24.7% of the 66,480 contigs used for binning). Of these MAGs, 45, 76 and 133 were defined in categories 1, 2 and 3 respectively. MAGs derived from multiple samples (in this case, from 42 faecal samples taken from 20 bank voles over two time points, and with two samples extracted twice – see Supplementary Information 1) often represent genomes of several taxa or strains, and further analysis and assembly will be required to generate complete bacterial genomes^43^. The remaining contigs, presumably representing a mixture of genetic material from the gut microbiota (such as viruses, plasmids, protists, some bacteria, *etc*.) as well as some read data derived from the bank vole host genome (potentially the fraction of highly repetitive DNA that could not be assembled in the draft genome) and also bank vole dietary material, are available for further analysis.

### Phylogenetic assessment of MAGs

To assess the phylogenetic diversity of the MAGs we predicted coding sequences in the secondary contigs (that had been assigned to draft genomes) using PRODIGAL^44^ v.2.6.3 (parameters: -m -p meta), and then searched for the sequences of a core panel of phylogenetic markers: the phylogenetic markers used are 16 ribosomal proteins (L2, L3, L4, L5, L6, L14, L16, L18, L22, L24, S3, S8, S10, S17 and S19) that are often syntenic, and which have been used for a comprehensive phylogenetic assessment of Bacteria, Archaea and Eukarya^45^, as well as phylogenetic placement of MAGs^34^. To provide wider context to the phylogenetic diversity of our MAGs, we also downloaded more than 1,600 Bacterial and Archaeal complete/representative genomes from NCBI’s Refseq database^46^ (https://www.ncbi.nlm.nih.gov/refseq/: date accessed 11/09/2019) (see Supplementary Table 4 for list of reference genomes). We searched for the 16 phylogenetic markers from our MAGs and the reference genomes using HMMSEARCH^47^ in HMMER v.3.1b2 (parameters: -E 1e-5) and the Hidden Markov Models for the 16 ribosomal proteins that were downloaded from Pfam database^48^ (https://pfam.xfam.org). Genomes that lacked half or more of the phylogenetic markers were not used in the phylogenomics analysis. Sequence data for each marker were aligned using MUSCLE^49^ v.3.8.31 (parameters: --maxiters 16). Alignments were trimmed using TRIMAL^50^ v.1.2rev59 (parameters: -automated1) and concatenated using the CONCAT script implemented by BINSANITY^38^. A detailed protocol for this phylogenomic workflow can be found at ProtocolsIO (10.17504/protocols.io.mp5c5q6). Following this protocol, we were able to construct a phylogenetic tree for 167 MAGs and 1,664 reference genomes using FASTTREE^51^ v2.1.9 (parameters: -gamma -lg), which was viewed and annotated using iTOL^52^ (https://itol.embl.de).

## Data Records

Project data (host metadata, metagenome shotgun sequence data, MAGs) have been deposited in NCBI Sequence Read Archive, under the SRA study accession SRP254056^31^. The concatenated alignment of the 16 phylogenomic loci for MAGs and reference genomes and the associated phylogenetic tree (Newick format) have been deposited with Figshare^53^.

## Technical Validation

Quality of the host-filtered and trimmed Illumina reads was quantified using FASTQC (https://www.bioinformatics.babraham.ac.uk/projects/fastqc/), and observed to be very good: example quality plots for six samples are given in Supplementary Figure 1, and the basic information on the total number of reads and other statistics associated with reads is presented in Supplementary Table 7. Potential cross-contamination of samples was limited by following guidelines for analyses of microbiota communities^54,55^; for example, DNA extractions took place within a dedicated laboratory space under a laminar flow hood using aseptic techniques (such as, surface sterilisation, use of sterile plasticware, and use of aerosol barrier pipette tips). Sample processing was completed within 10 days by AL, using the same batch of DNA isolation kits for all samples. DNA concentrations were quantified with a Qubit 2.0 fluorometer (Invitrogen, Carlsbad, CA, USA) and NanoDrop Spectrophotometer (ThermoFisher Scientific, MA, USA). Extracting total DNA from 0.1 g of faecal material using the PowerFecal DNA Isolation kit (MoBio Laboratories, Carlsbad, CA, USA) typically provided 20–100 ngµl^−1^ DNA (Supplementary Table 2) for library construction. To assess for potential contamination (*e.g*. from laboratory reagents, and/or contamination of samples), we extracted DNA from two samples twice, independently, and these samples were sequenced as separate libraries also (*i.e*. there were 42 libraries prepared) (Supplementary Table 1). We found low (*r* < 0.2) correlations (Supplementary Table 6) in the abundances of the MAGs among most pairs of samples (see Supplementary Table 5 for mean coverages per sample and MAG, which were calculated using ANVI’O v.5.2^39,40^ as the ‘*mean coverage of a contig divided by overall sample mean coverage*’). Hence, these MAGs exhibit substantial variation in their abundance among individuals and timepoints (Supplementary Table 5), which is consistent with expected levels of inter-individual and temporal variation in rodent gut microbiota community composition^14,16–18^. The notable exceptions to these low pairwise correlations in MAG abundance were the comparisons of MAG abundances between the pairs of independent, replicate DNA extractions (*r* = 0.999 for samples 25 and T25, and *r* = 1.000 for samples 69 and T96) (Supplementary Table 6). The high similarity in MAG abundance among the independent, replicate extractions, but otherwise individual community profiles (that are typical rodent gut microbiota^13–17^) in each sample, implies that read data reflect the sample’s microbiota and that contamination of samples during DNA extraction was minimised^55^.

Metagenome data have been assembled and refined into MAGs using the automated quality control steps, assembly procedures and the thresholds described in the manuscript. In addition, contigs were passed through NCBI’s Contamination Screen to remove any residual adaptor contamination (21 out of 16,486 contigs contained within the MAGs had one NGS adaptor sequence that was removed; Supplementary Table 3). Technical validation of the taxonomic assignments, completeness and potential contamination of the putative bacterial MAGs was achieved using several softwares, but we recommend that further, independent technical validation be applied by users of these draft MAGs.

## Supplementary information

Supplementary Figure 1

Supplementary Tables 1-6
