# Supplementary material for: Two hundred and fifty-four metagenome-assembled bacterial genomes from the bank vole gut microbiota
Source: Sci Data. 2020 Sep 23;7:312. doi: 10.1038/s41597-020-00656-2 (PMC7511399; doi:10.1038/s41597-020-00656-2)
Supplement: Supplementary file 1 — Supplementary Figure 1 [file 41597_2020_656_MOESM1_ESM.pdf]

Sample 146

Per base sequence quality

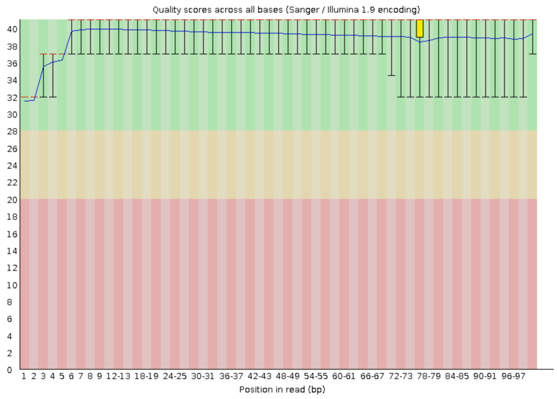

Per base sequence quality

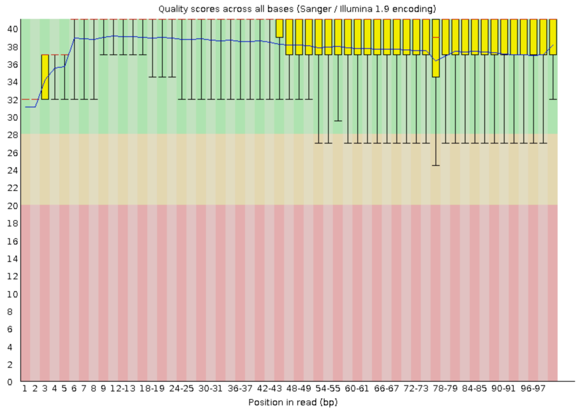

Sample186

Per base sequence quality

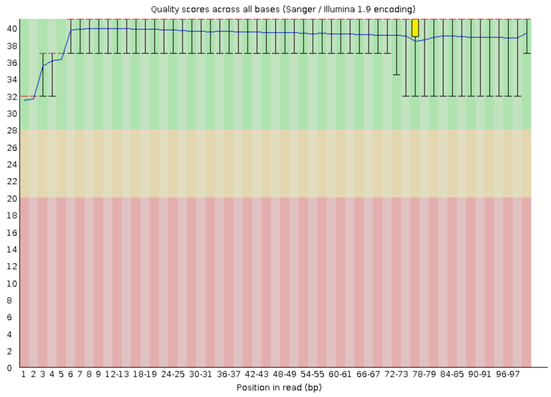

Per base sequence quality

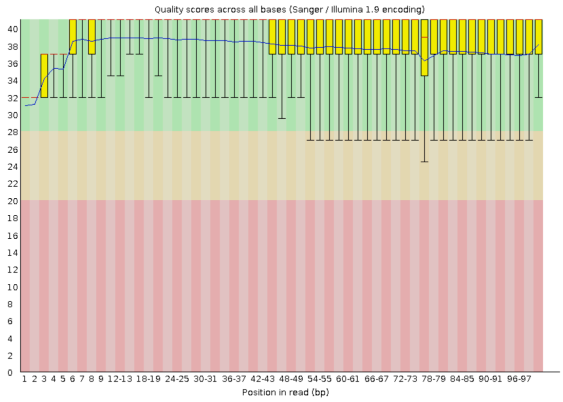

Sample189

Per base sequence quality

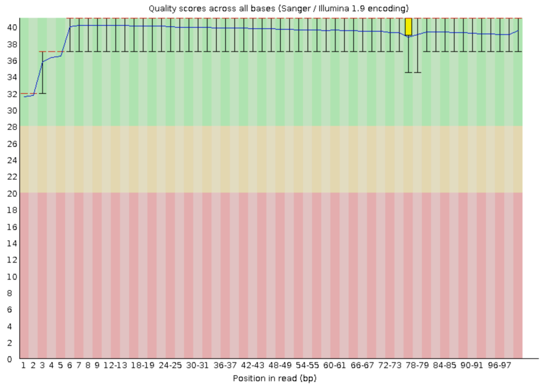

Per base sequence quality

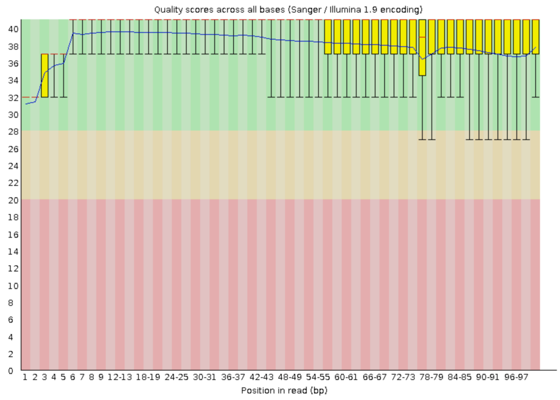

Sample 218

Per base sequence quality

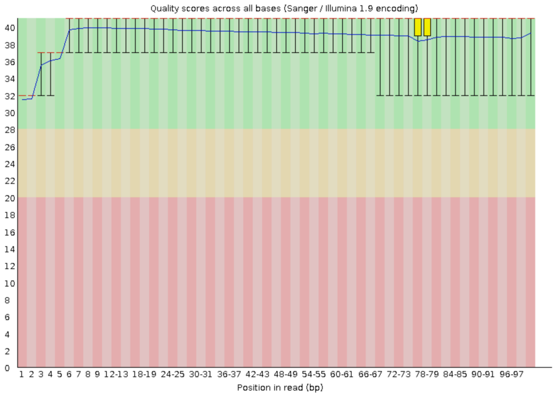

Per base sequence quality

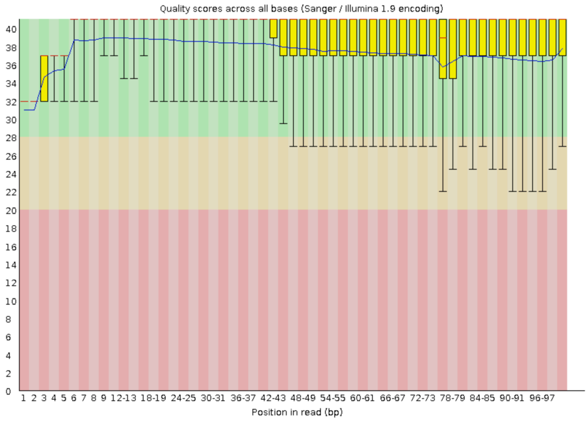

Sample248

Per base sequence quality

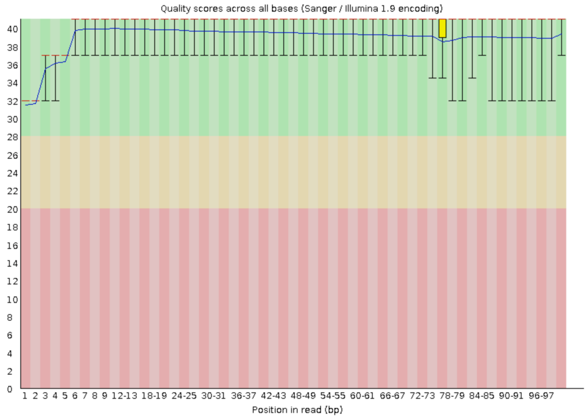

Per base sequence quality

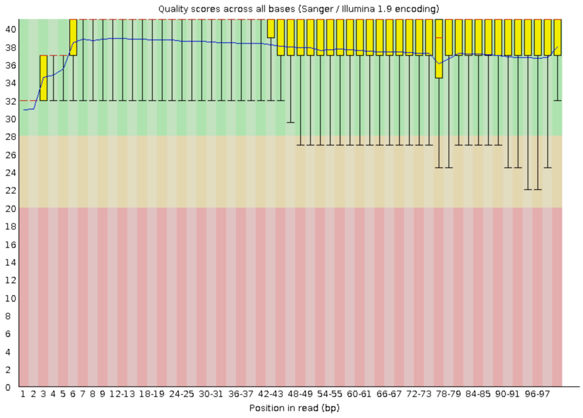

Sample 249

Per base sequence quality

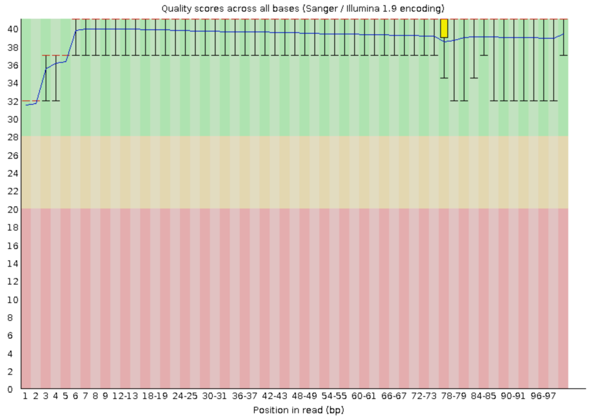

Per base sequence quality

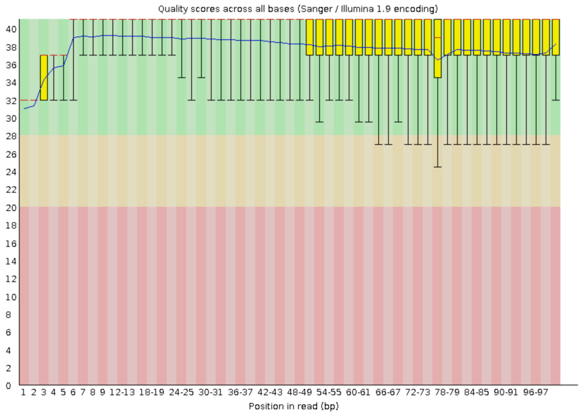

Supplementary Figure 1. Example output (for six samples of paired end sequence data) from FASTQC that summarises the per base sequence quality of read data (from bank vole faecal meta genome sequencing) that have passed through host-filtering and quality trimming procedures.
